# Post feline infectious peritonitis progressive hydrocephalus: a case series

**DOI:** 10.3389/fvets.2025.1694679

**Published:** 2025-11-17

**Authors:** Mallory Clouse, W. Ryan Detwiler, Erika A. Gibson, Jessica Eich, Jason Berg, Richard Joseph

**Affiliations:** 1 Department of Neurology, MedVet Cleveland, Cleveland, OH, United States; 2 Department of Neurology, Guardian Veterinary Specialists, Brewster, NY, United States; 3 Department of Radiology, BluePearl Pittsburgh, Pittsburgh, PA, United States

**Keywords:** feline infectious peritonitis (FIP), hydrocephalus, ventriculoperitoneal (VP) shunt, feline coronavirus (FCoV), vestibular function, MRI

## Abstract

**Background:**

While in recent years, great strides have been made in treating cats with feline infectious peritonitis (FIP), long-term sequelae of the disease are unknown.

**Objectives:**

We aimed to describe the MRI findings of cats who were presented for follow-up care post-treatment with GS-441524.

**Animals:**

Four cats who underwent treatment for FIP diagnosed based on MRI and were re-presented 13–15 months post-treatment were evaluated. Three cats were re-presented with vestibular signs.

**Methods:**

Cases were selected based on clinical signs and history of treatment of neurologic FIP. All cats were examined by a veterinary neurologist or neurology resident at each appointment. All MRIs were reviewed by a board-certified veterinary radiologist. Necropsy of Cat 2 was performed by a board-certified veterinary pathologist. Details of diagnostic test results and treatment were retrospectively acquired by review of medical records. Cerebrospinal fluid (CSF) analysis was performed and interpreted by a board-certified veterinary pathologist at an outside reference laboratory. FCoV PCR testing was submitted to university reference and commercial reference laboratories.

**Results:**

All cats presented had been treated with owner-sourced GS-441524 (off-label) for the standard 84 days. All cats improved clinically after treatment, evidenced by neurologic exam findings and owner reports. Three affected cats were re-presented for neurologic decline >1 year post-treatment; one cat remained neurologically stable. After MRI confirmed progressive hydrocephalus in the three affected cats, all were treated with ventriculoperitoneal (VP) shunt placement, with noted improvement in neurologic status post-operatively. The fourth cat showed improvement of hydrocephalus on MRI based on ventricular size. CSF analysis was performed for all cats, showing no evidence of active inflammation. CSF of three cats were negative for FCoV PCR (one cat had insufficient quantity collected for testing). One affected cat was euthanized for recurrent urinary obstruction; necropsy showed moderate to severe hydrocephalus with mild parenchymal collapse, and no evidence of active infection or inflammation was noted.

**Conclusion and clinical importance:**

Progressive post-FIP hydrocephalus should be a differential for cats re-presenting with neurologic signs after treatment for FIP with GS-441524. As the treatment is now legally available in the United States, more cases are likely to occur. It is important to differentiate these progressive post FIP hydrocephalus cases from relapses of FIP, as prognosis and treatment differ.

## Introduction

Feline infectious peritonitis (FIP) is the most common cause of neurologic dysfunction in young cats (1, 2). Cats with neurologic FIP exhibit a spectrum of abnormalities including ataxia, behavior changes, and vestibular dysfunction (1–5). While FIP is difficult to diagnose, some signs are visible via magnetic resonance imaging (MRI) for diagnosis of neurologic FIP, specifically ventriculomegaly and periventricular contrast enhancement, indicating ventriculitis (6–8). Historically, FIP was considered fatal; recently, treatment with GS-441524 has proven successful (3, 8–13). Before 2024, in the United States, treatment for FIP was unlicensed (10). In June 2024, the United States Food and Drug Administration (FDA) approved one source of the medication, making it commercially available. While there is a growing population of FIP survivors, long-term sequelae are unknown. Relapse is possible and may be more common in non-effusive FIP (3).

Because effective treatment for FIP is more widely available, it is important to document and recognize potential long-term complications. To our knowledge, these case reports represent the first documented instances of progressive hydrocephalus following treatment with GS-441524, with MRI conducted before and after treatment, treated with ventricular-peritoneal shunt placement. Concurrently, Case 4 highlights a cat showing improvement of ventriculomegaly on MRI, supporting that development of progressive hydrocephalus post-FIP treatment affects a variable number of cats.

## Case reports

### Case 1

A 14-month-old, male, neutered, domestic shorthair (DSH) cat was presented to an emergency clinic for generalized tremors, anorexia, and lethargy. Bloodwork showed elevated globulins and a decreased albumin-globulin ratio (A:G ratio; See Table 1). FIP 7B ELISA was high (Table 1), prompting a presumptive diagnosis of FIP. He was prescribed a 15-day taper of prednisolone starting at ~2 mg/kg BID (twice daily), and, 2 days later, the owners sourced GS-441524 from a third party and started 5 mg/kg injections subcutaneously BID. Appetite and demeanor improved with treatment, but he was reported to be ataxic and was referred to neurology for further diagnostics and treatment 1 week later.

**Table 1:** Summarizes the exam and diagnostic findings for cases 1–4.

**Case**	**Cat 1**	**Cat 2**	**Cat 3**	**Cat 4**
Initial clinical signs (reported by owner)	Lethargy, hyporexia, tremors	Weight loss, hyporexia, crouched posture, lethargy	Progressive ataxia (initially responsive to steroids), lethargy	Gait change, lethargy, hyporexia
Bloodwork abnormalities	WBC 17.41 (2.87–17.02 K/μl) Neutrophils 14.43 (2.30–10.29 K/μl) Total protein 11.5 (5.7–8.9 g/dl) Globulin 8.3 (2.8–5.1 g/dl) Albumin:globulin ratio 0.4 FIP 7b ELISA ≥ 1:320 (< 1:40)	Platelet count 169 (200–500 10^3^/μl)	No significant findings	Albumin:globulin ratio 0.6 FIP 7b ELISA 1:160 (< 1:40)
Initial neurological exam	Left-sided head tilt Vestibular ataxia Delayed postural reactions (left more than right)	Obtunded mentation Vertical downbeat nystagmus Dysmetric in thoracic limbs, truncal sway, increased tone thoracic limbs Delayed postural response in left greater than right thoracic limb	Obtunded mentation Non-ambulatory tetraparesis with minimal motor Thoracic limb extensor rigidity with opisthotonus. Postural responses delayed in all limbs	Pelvic limb proprioceptive ataxia with no postural deficits
Neurolocalization (initial presentation)	Central vestibular (left more than right)	Central vestibular (left more than right)	Diffuse brainstem	T3–L3
Initial MRI findings	Diffuse ventriculomegaly with concurrent dilation of the central canal A T2 heterogeneously hyperintense lesion along the rostral fourth ventricle caudal to the mesencephalic aqueduct R>L cerebellum exhibited varied hyperintensity on T2/FLAIR Focal meningeal enhancement along the brainstem	Severe dilation of the fourth and lateral ventricles Moderate dilation of the central canal of the included cranial cervical spinal cord Mild to moderate ependymal enhancement of the fourth ventricle and both lateral ventricles	Diffuse ventriculomegaly with associated dilation of the mesencephalic aqueduct, with concurrent central canal dilation of the included cervical spinal cord Moderate focal meningeal enhancement of the brainstem and circumferential enhancement of all ventricles and the midbrain aqueduct	TL–unremarkable study Brain–diffuse ventriculomegaly with associated severe bilateral dilation of the olfactory recesses of the lateral ventricles Mild diffuse periventricular FLAIR hyperintensity Caudoventral transtentorial herniation of the colliculi, with concurrent coning and herniation of the caudal cerebellum through the foramen magnum
Time between diagnosis and re-presentation to specialty hospital	14 months	16 months	16 months	16 months
Reason for re-presentation	Wide-based stance, reluctance to jump	Progressive head tilt, worsening ataxia	Falling over, worsening vestibular ataxia	1 year recheck (routine)
Recheck neurologic exam	Slight left head tilt Delayed postural response (right sided)	Obtunded mentation Right sided head tilt, positional vertical nystagmus Non-ambulatory tetraparesis Increased tone in left thoracic limb Decreased postural response in left greater than right thoracic limb	Positional vertical nystagmus Non-ambulatory due to vestibular ataxia, falling to right Decreased postural response in left thoracic limb	Normal neurologic exam
Neurolocalization (recheck)	Central vestibular (right more than left)	Central vestibular (left more than right)	Central vestibular (left more than right)	NA
Re-check MRI findings	Progressive diffuse ventriculomegaly without contrast enhancement	Progressive diffuse ventriculomegaly without enhancement of the ependymal lining Marked T2 hyperintensity of cervical spinal cord	Progressive diffuse ventriculomegaly with mild contrast enhancement of the ventral portion of the lining of the fourth ventricle	Persistent diffuse ventriculomegaly with mild improvement in lateral ventricle size
CSF analysis	No cytologic abnormalities QNS for fluid analysis	No cytologic abnormalities Prot 40.8 mg/dl (0.0–36.0) Nuc cell count 0 cell/μl RBC 0 cell/μl	No cytologic abnormalities QNS for fluid analysis	No cytologic abnormalities Prot 18.7 mg/dl (0.0–36.0) Nuc cell count 1 cell/μl (0–4) RBC 4 cell/μl (0–30)
FCoV PCR (CSF)	Negative	Negative	NA	Negative
Post VPS neurologic exam	Equivocal vestibular ataxia with no head tilt	Ambulatory with vestibular ataxia Persistent increased tone in the left thoracic limb	Mildly decreased postural response in the left thoracic limb	NA
Necropsy	NA	Urethral obstruction Marked dilation of lateral, third, and fourth ventricles (gross and histologically). Periventricular gitter cells (few) and glial cells (rare) No significant inflammatory cell infiltrate or cellular degeneration is observed in the tissue sections examined FIP IHC negative	NA	NA

WBC, white blood cells; RBC, red blood cells; ELISA, enzyme-linked immunosorbent assay; FLAIR, fluid attenuated inversion recovery; QNS, quantity not sufficient; IHC, immunohistochemistry.

On presentation, he showed a left sided head tilt, vestibular ataxia, and decreased postural reactions, which were more pronounced on the left side. He was admitted for MRI, which showed hydrocephalus and inflammatory changes consistent with neurologic FIP (Table 1). Due to evidence of increased intracranial pressure and confidence in diagnosis of FIP due to MRI findings, clinical signs, and bloodwork findings supportive of FIP, cerebrospinal fluid (CSF) analysis was not performed. He recovered uneventfully from anesthesia. The 84-day course of GS injections was completed without complications. A follow-up neurological exam 1 month later showed resolved head tilt, mild vestibular ataxia, and mild left-sided postural deficits.

**Figure 1:**
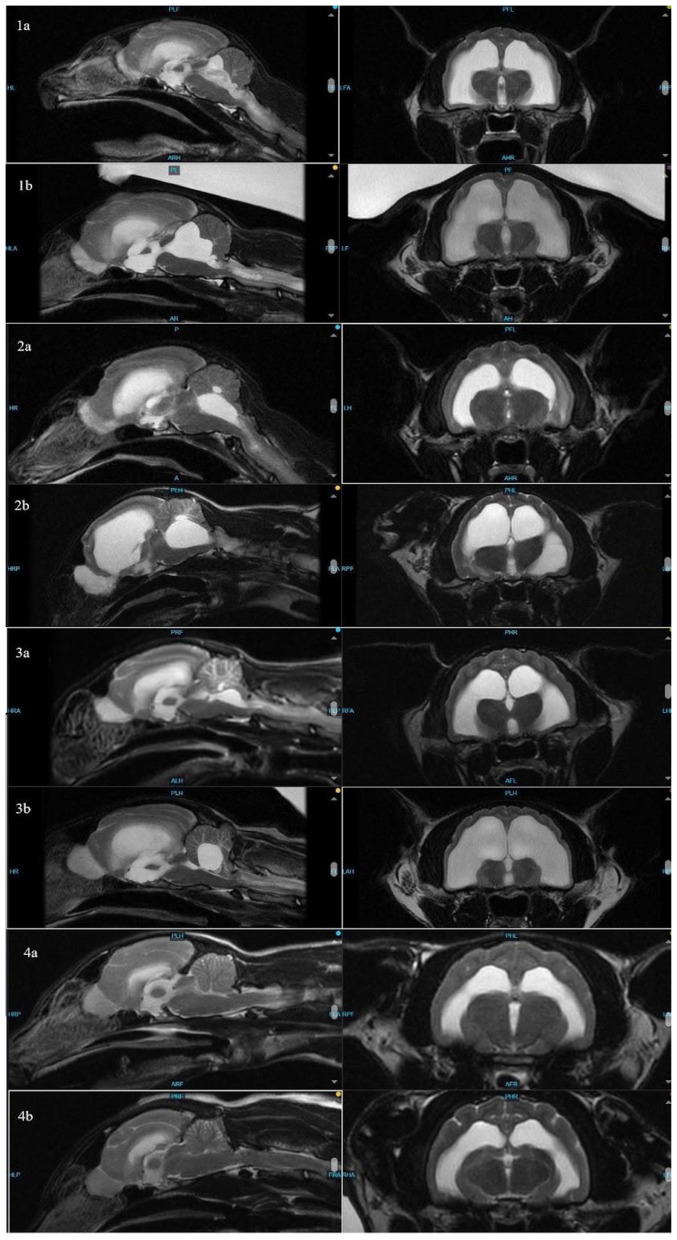
Sagittal **(left)** and transverse **(right)** images of cats 1–4 to compare ventricle sizes and hydrocephalus at time of FIP diagnosis **(a)** and re-presentation **(b)**.

The cat was re-presented (aged 31 months) due to vestibular signs 13 months later. On examination, the cat was noted to have a mild left head tilt, decreased postural reactions on the right side, and pronounced vestibular ataxia. Repeat brain MRI showed markedly progressive hydrocephalus with no evidence of active inflammation (Figure 1). Due to progressive hydrocephalus and declining neurologic status, a ventriculoperitoneal shunt (VPS) was recommended. A Codman programmable VPS, with pressure set at 100 cm H2O (centimeters of water), was placed in the right lateral ventricle without complication; during surgery, CSF was sampled and submitted for analysis and Feline Coronavirus (FCoV) polymerase chain reaction (PCR). CSF collected was not sufficient for fluid analysis; cytology showed no evidence of inflammation. FCoV PCR was negative. The cat was discharged 2 days following shunt placement. At the time of discharge, he remained ataxic but was able to ambulate. He was re-evaluated 2 weeks after shunt placement, at which time he was noted to have mild vestibular ataxia, a subtle left head tilt, and subtle right-sided postural deficits. He presented 3 months post-shunt placement for his final recheck; the only neurologic deficit noted was equivocal vestibular ataxia. Telephone communication with the client 1 year after shunt placement indicated he was doing well at home, and he is alive at the time of writing.

### Case 2

A 14-month-old, male, neutered, DSH was presented to the neurology department for evaluation of episodes of non-distractable self-mutilation and aggression. Complex partial seizures were suspected, and episode frequency and severity were improved with phenobarbital 2.2 mg/kg BID.

After 2 months, he was re-presented for behavior changes and ataxia. On examination, the cat was obtunded, non-ambulatory, had resting vertically downbeat nystagmus, truncal sway, and decreased postural reactions on the left (Table 1). Brain MRI showed hydrocephalus and ependymal enhancement consistent with FIP. He was prescribed dexamethasone at 0.1 mg/kg BID for 2 weeks, tapering over 6 weeks. After 2 weeks, his owners elected to start treatment with GS-441524 injections for 50 days (5 mg/kg subcutaneously BID), switching to oral capsules (10 mg/kg/day) for the remaining 34 days due to difficulty giving injections. At his visit at the end of the treatment course, he showed left-sided vestibular ataxia but was able to walk without support.

The cat was re-presented 14 months post treatment (aged 33 months) for yearly seizure management, and his owners reported that he was having difficulty walking at home. At re-presentation, neurologic exam revealed positional vertical downbeat nystagmus, right-sided head tilt, thoracic limb postural deficits, and vestibular ataxia. Repeat brain MRI showed marked progression of hydrocephalus (Table 1). VPS was recommended but declined in favor of medical management. After being discharged, the cat suffered a urinary obstruction and was unblocked at another facility. He was managed medically for hydrocephalus with prednisolone at 0.6 mg/kg once daily and was re-presented ~2 months later for VPS placement. A Codman programmable VPS (pressure set at 100 cm H2O) was placed in the left ventricle without complication. CSF showed mild blood contamination with no nucleated cells. FCoV PCR was negative. Postoperatively, the cat showed significant improvement in gait and balance, although he continued to have significant vestibular ataxia. He was re-presented several times following VPS due to progressive worsening of ataxia and vestibular signs, which prompted the need for shunt pressure adjustments (decreased by 10–20 cm H2O). Notably, he showed a positive response to a reduction in shunt pressure (Supplementary Image 1). After 3 months, he was re-presented for urinary obstruction and was admitted for cystotomy to remove cystoliths. During surgery, the shunt was flushed and confirmed functional. CSF collected showed elevated protein without inflammatory cells. Shunt pressure was lowered to 30 cm H2O (lowest setting). Following the procedure, the owners reported a significant improvement in mobility. After 4 months, he was humanely euthanized due to complete urinary obstruction. Necropsy confirmed no active FIP infection nor obvious cause for hydrocephalus (Table 1).

### Case 3

A 10-month-old, male, neutered, Maine Coone cat was presented for lethargy and ataxia, which had progressed despite treatment with prednisolone at 1 mg/kg/day for 1 week. On initial neurologic exam, the cat was non-ambulatory with thoracic limb extensor rigidity and minimal motor function in all limbs. He had episodes of opisthotonus as well as episodes of squinting and cervical fasciculations suspected to be secondary to pain. Brain MRI showed diffuse ventriculomegaly and meningeal enhancement consistent with FIP (Table 1). He was treated with an 84-day course of injections of GS-441524 at a dose of 10 mg/kg/day, as well as a prednisolone taper at 1 mg/kg/day, tapering over 3 weeks. His neurological exam was normal 2 weeks after finishing the GS-441524 injections.

At 26 months old, the cat was presented through the emergency department for concerns of recurrence of FIP based on a week-long history of progressive vestibular ataxia. Prior to transfer to neurology, he was given a single dose of GS-441524 (10 mg/kg) due to concern of relapse. Neurologic examination showed positional vertical nystagmus, decreased postural reactions in the left thoracic limb, and falling to the right. The cat was non-ambulatory due to vestibular ataxia. Repeat brain MRI showed progressive hydrocephalus (Figure 1). CSF was not collected due to cerebellar herniation through the foramen magnum. Initially, the owners opted for medical management with dexamethasone (0.1 mg/kg/day) and omeprazole (10 mg/kg BID), but his neurologic status declined when the dexamethasone was tapered.

At 2-week post-MRI recheck, he was ambulatory with normal postural reactions but still had mild left vestibular ataxia with positional vertical nystagmus. A total of 3 months post diagnosis, his neurologic status had declined, and he was non-ambulatory with a marked left-sided head tilt. His owners elected for VPS placement. A 100 cm H2O Codman fixed pressure VPS was placed in the left lateral ventricle. CSF collected intraoperatively showed no cytologic abnormalities; quantity was not sufficient for FCoV PCR. He was prescribed a dexamethasone taper of 0.1 mg/kg daily, tapering over 9 days. The day following surgery, the cat was able to ambulate independently, and his head tilt had improved (Supplementary Image 2). At 2-week recheck, the cat was off medications and was neurologically normal except for mildly delayed postural reactions in the right thoracic limb (suspected to be secondary to left VPS placement). At the time of writing (8 months post-operatively), the cat is reported to be doing well at home.

### Case 4 (control)

A 7-month-old, female, spayed, DSH cat was presented for evaluation of progressive pelvic limb ataxia over 2 to 3 weeks. On examination, the cat was ataxic in the pelvic limbs without postural deficits. MRI of the thoracolumbar spine was performed, which was unremarkable, and sagittal scans of the brain were abnormal, so a brain study was performed. MRI showed diffuse ventriculomegaly with periventricular hyperintensity consistent with FIP (Table 1). She was prescribed prednisolone at 1 mg/kg/day, tapering over 2 weeks. She wasstarted on GS-441524 injections at 10 mg/kg for 84 days. She was re-evaluated 2 weeks after finishing the GS course, and her neurologic exam was normal.

She was presented again 15 months later (aged 25-months) for yearly monitoring. While she had recovered well and was clinically normal at home, her initial MRI had been abnormal (Table 1). With the history of two cats (Cases 1 and 2) with neurologic FIP treated prior to her returning for progressive neurologic signs, and the subsequent changes in those MRIs, and lack of consensus regarding long-term follow-up for neurologic FIP cases, recheck MRI and CSF was offered for monitoring for this cat. The re-checking neurological exam was unremarkable. Compared to her previous MRI, recheck brain MRI showed mildly improved ventriculomegaly based on the lateral ventricle to brain height ratio (Table 2). CSF cytology and fluid analysis were unremarkable. FCoV PCR performed on spinal fluid was negative.

**Table 2:** Ventricle to brain height ratio of cats 1–4, demonstrating progressive hydrocephalus in cats 1–3 and stable ventricle size in cat 4.

**Case**	**Brain height**	**Ventricle height [L]**	**Ventricle height [R]**	**Vh/BH L (%)**	**Vh/BH R (%)**
Cat 1a	31.37 mm	11.84 mm	11.67 mm	37.74%	37.20%
Cat 1b	33.00 mm	17.29 mm	16.97 mm	52.40%	51.42%
Cat 2a	33.71 mm	9.96 mm	11.53 mm	29.55%	34.20%
Cat 2b	34.93 mm	12.89 mm	17.58 mm	36.73%	50.33%
Cat 3a	31.81 mm	9.69 mm	10.12 mm	30.46%	31.81%
Cat 3b	33.88 mm	16.90 mm	17.23 mm	49.88%	50.86%
Cat 4a	29.67 mm	7.41 mm	7.60 mm	24.97%	25.62%
Cat 4b	29.74 mm	6.18 mm	6.74 mm	20.78%	22.66%

## Discussion

This report details three cases of cats with progressive hydrocephalus after clinical recovery from feline infectious peritonitis. All affected cats were re-presented for neurological evaluation for concerns of decline more than 1 year after completing treatment courses for FIP. An unaffected cat was presented for routine monitoring after treatment.

All affected cats were presented 13–14 months after completing GS-441524 treatment. The neurologically normal cat had a repeat MRI 15 months after treatment. All cats showing vestibular signs had resolution or near resolution of ventriculitis based on pre- and post-contrast sequencing and progressive hydrocephalus based on comparison of pre- vs. post-treatment MRIs, including brain height to lateral ventricle height ratios (14). Brain height to lateral ventricular ratio has been used to estimate cerebral ventricle volume in cats and has been used to quantify feline hydrocephalus (14, 15). Although the caseload is too limited to determine statistical significance, this ratio, when applied to these cases, does suggest progressive ventriculomegaly in affected cats (Table 2).

To date, no consensus exists for long term monitoring for cats which have recovered from FIP. Relapse of feline infectious peritonitis usually occurs within 60–84 days of completing treatment, although there have been reports of relapse up to 450 days after stopping treatment (11, 16). For cats presenting with central vestibular signs more than 1 year after treatment, MRI should be recommended. Case 4 had no progressive neurologic signs, and repeat MRI showed mild improvement in ventriculomegaly, supporting that follow-up MRI in cases that are clinically normal may not be necessary.

It is unclear why affected cats developed progressive hydrocephalus. Based on imaging (near complete resolution of contrast uptake and FLAIR periventricular hyperintensity), clinical signs, and CSF analysis, none of the cats experienced relapse of neurologic FIP. Negative FCoV PCR on CSF supports that the cats had no active FIP infection. FCoV PCR on CSF of cats with neurologic or ocular FIP demonstrates 100% specificity; however, the sensitivity is 85.7%, indicating that a negative FCoV PCR does not rule out FIP (17). Necropsy of Cat 2 confirmed no active inflammation of nervous tissue; the ventricular system was unremarkable. Further, IHC of brain tissue for FIP was negative, which has sensitivity of 97%−100% and specificity of up to 100%; this supports that Cat 2 had cleared infection (18). Therefore, another mechanism of hydrocephalus is considered most likely.

Several mechanisms for hydrocephalus are reported in cats including skull shape (as for Persian and other brachycephalic cat breeds), stenosis of the mesencephalic aqueduct (due to congenital malformation, inflammation, or infiltrative disease), blockage of the lateral apertures, and inflammation of the ventricles/periventricular areas (7, 19, 20). All cats represented in this report were mesocephalic. While congenital hydrocephalus, as well as hypertensive hydrocephalus, are expected to progress with age, the decrease in ventricular size in Cat 4 does not support these mechanisms as a cause of the clinical presentation for affected cats (20). Inflammation of the periventricular area is hypothesized to be how FIP infection causes hydrocephalus, but no active inflammation was supported by CSF cytology or MRI findings in these patients.

In human medicine, a condition called post-infective or post-inflammatory hydrocephalus has historically been associated with infections such as tuberculosis and bacterial meningitis, however, cases of post-inflammatory hydrocephalus associated with neurologic COVID-19 infection have been reported (21–23). Hence, there is early evidence that a coronavirus could result in post-inflammatory hydrocephalus. While the mechanism of post-inflammatory hydrocephalus is not completely understood, it is suggested to result from a combination of increased CSF secretion from the choroid plexus secondary to inflammatory cytokines and decreased CSF absorption secondary to scarring (21). Although neither gross nor microscopic abnormalities of the ventricular system in the post-mortem were identified in Cat 2, it is possible that identifying such abnormalities would require more advanced testing or only be identifiable at magnifications higher than those used for routine necropsy. A combination of increased CSF secretion and decreased absorption seems the most probable cause for the affected cats, but further studies are needed.

Extrapolating from human data, the frequency of post-inflammatory hydrocephalus would be expected to be low (21). As more cats are treated for FIP, the frequency of this condition and potential risk factors may be better characterized. While all affected cats in this case series were male, this is likely selection bias due to low case numbers.

**Figure 2:**
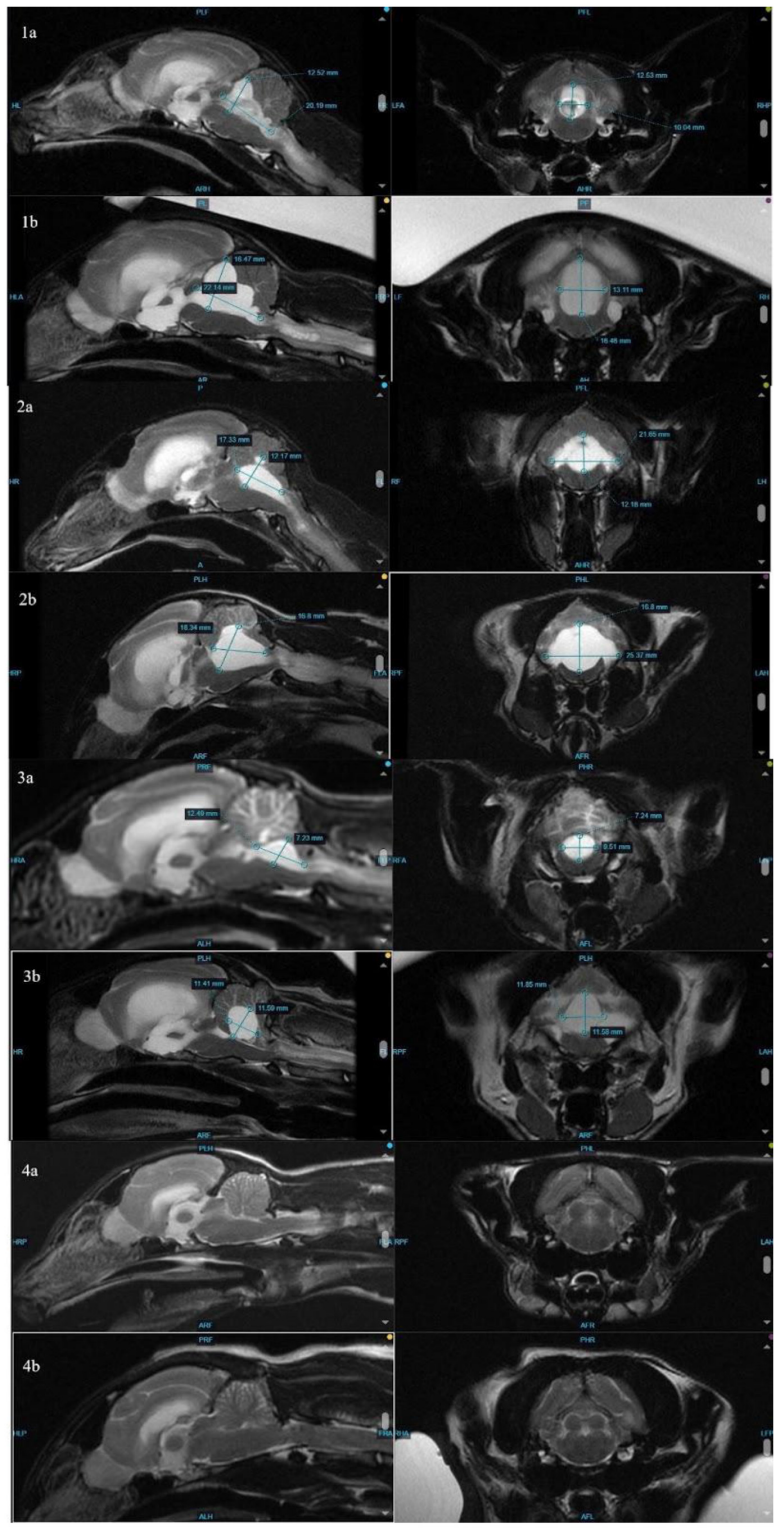
Comparison of measurements (height, width, and length) of the fourth ventricle at initial MRI **(1–4a)** and repeat MRI **(1–4b)**. Note that the fourth ventricle in cat 4 (clinically normal at time of follow-up MRI) is too small to measure.

The distribution of fluid in the ventricles, with the disproportionate enlargement of the fourth ventricle, would explain clinical signs of vestibular dysfunction seen in these cases due to compression of the cerebellum and brainstem. While lateral ventricle size and its relation to brain height has been studied to evaluate clinical hydrocephalus in cats, there is a paucity of veterinary literature classifying fourth ventricular size, especially in cats (14). In dogs, although the size of the fourth ventricle is found to be statistically different between hydrocephalus and ventriculomegaly groups, it is suggested that body mass may affect size of the fourth ventricle (24). Individually, each affected cat in this series presented with increased size in all ventricles, including, most dramatically, the fourth ventricle. In contrast, Case 4 showed no discernible enlargement of the fourth ventricle pre- or post-treatment (Figure 2). While there is no standardized ratio or measurement that has been proven to characterize the size or volume of the fourth ventricle in cats, in all affected cats, the fourth ventricle displays progressive dilation.

In human literature, a disproportionately large communicating fourth ventricle (DLCFV) is a rare condition in which a patient with communicating hydrocephalus exhibits marked enlargement of the fourth ventricle (25). These patients have history of disease affecting the caudal fossa and present with cerebellovestibular signs (25). Ventriculoperitoneal shunt placement is the treatment of choice for post-inflammatory hydrocephalus and has been anecdotally reviewed for DLCFV in people (23, 25). While limited case reports suggest that VPS placement may not be efficacious for DLCFV in people, this is suggested to be due to use of a programmable shunt system with an anti-siphon device that was unable to lower the pressure in the fourth ventricle appropriately (as very low to negative pressure is required) (25). However, ventriculoperitoneal shunts that can achieve these lower pressures (either without an anti-siphon device or with features that allow pressure to change with inspiration) can successfully drain the fourth ventricle and provide symptom relief (25). While all cats responded to VPS placement, one of the cats with a programmable shunt showed evidence of needing repeated pressure changes (decreasing pressure) which could represent a phenomenon similar to what has been described in people. While there is not enough data to support one type of shunt over another for cats with the described condition, studies evaluating the outcomes of programmable vs. fixed-pressure shunt cases may be needed in the future. The cases presented support that VPS placement is viable for cats with progressive hydrocephalus post FIP treatment.

Limitations of this paper include low case numbers. As more cats are treated for FIP, it is likely a larger caseload can be compiled. With a larger caseload, potential risk factors for the development of this condition may be identified. Additionally, there is a lack of long-term imaging follow-up in FIP cases for cats that are clinically normal, so trends regarding ventricle size and ventricle to brain height ratio are not established. Further investigation into treatment outcomes, specifically comparing outcomes of cats with fixed pressure shunts vs. programmable shunts, may be helpful in developing more standard treatment for this condition. Tissue studies to evaluate potential changes in the characteristics of the choroid plexus in affected cases may be useful to clarify the pathophysiology of the development of post-FIP hydrocephalus.

## Data Availability

The original contributions presented in the study are included in the article/supplementary material, further inquiries can be directed to the corresponding author.

## References

[1] Bradshaw JM Pearson GR Gruffydd-Jones TJ. A retrospective study of 286 cases of neurological disorders of the cat. J Comp Pathol. (2004) 131:112–20. doi: 10.1016/j.jcpa.2004.01.010 15276850 PMC7134559

[2] Crawford AH Stoll AL Sanchez-Masian D Shea A Michaels J Fraser AR . Clinicopathologic features and magnetic resonance imaging findings in 24 cats with histopathologically confirmed neurologic feline infectious peritonitis. J Vet Int Med. (2017) 31:1477–86. doi: 10.1111/jvim.14791 28833469 PMC5598904

[3] Pedersen NC. The Neurological Form of Feline Infectious Peritonitis and GS-441524 Treatment. Davis, CA: UC Davis Center for Companion Animal Health (2021). p. 7.

[4] Timmann D Cizinauskas S Tomek A Doherr M Vandevelde M Jaggy A. Retrospective analysis of seizures associated with feline infectious peritonitis in cats. J Feline Med Surg. (2008) 10:9–15. doi: 10.1016/j.jfms.2007.06.004 17765591 PMC7128422

[5] Hoey C Nye G Fadda A Bradshaw J Barker EN. Subarachnoid diverticulum associated with feline infectious peritonitis in a Siberian cat. J Feline Med Surg Open Rep. (2020) 6:2055116920941477. doi: 10.1177/2055116920941477 33149927 PMC7580156

[6] Rissi DR. A retrospective study of the neuropathology and diagnosis of naturally occurring feline infectious peritonitis. J Vet Diagn Invest. (2018) 30:392–9. doi: 10.1177/1040638718755833 29411701 PMC6505806

[7] Foley JE Lapointe JM Koblik P Poland A Pedersen NC. Diagnostic features of clinical neurologic feline infectious peritonitis. J Vet Int Med. (1998) 12:415–23. doi: 10.1111/j.1939-1676.1998.tb02144.x 9857333 PMC7167019

[8] Dickinson PJ Bannasch M Thomasy SM Murthy VD Vernau KM Liepnieks M . Antiviral treatment using the adenosine nucleoside analogue GS-441524 in cats with clinically diagnosed neurological feline infectious peritonitis. J Vet Int Med. (2020) 34:1587–93. doi: 10.1111/jvim.15780 32441826 PMC7379040

[9] Pedersen NC. History of Feline Infectious Peritonitis 1963-2022–First Description to Successful Treatment. Vol. 944. Davis, CA: Center for Companion Animal Health; School of Veterinary Medicine; University of California (2022). p. 201963–2022.

[10] Jones S Novicoff W Nadeau J Evans S. Unlicensed GS-441524-like antiviral therapy can be effective for at-home treatment of feline infectious peritonitis. Animals. (2021) 11:2257. doi: 10.3390/ani11082257 34438720 PMC8388366

[11] Taylor SS Coggins S Barker EN Gunn-Moore D Jeevaratnam K Norris JM . Retrospective study and outcome of 307 cats with feline infectious peritonitis treated with legally sourced veterinary compounded preparations of remdesivir and GS-441524 (2020–2022). J Feline Med Surg. (2023) 25:1098612X231194460. doi: 10.1177/1098612X231194460 37732386 PMC10812036

[12] Nekouei O St-Hilaire S Hui PC Chan K Chan IS Ngan SYL . Potential therapeutic effects of GS-441524 and GC376 in cats with feline infectious peritonitis. Vet Evid. (2022) 7:2–12. doi: 10.18849/ve.v7i1.522 PMC13011082 42007291

[13] Zwicklbauer K Krentz D Bergmann M Felten S Dorsch R Fischer A . Long-term follow-up of cats in complete remission after treatment of feline infectious peritonitis with oral GS-441524. J Feline Med Surg. (2023) 25:1098612X231183250. doi: 10.1177/1098612X231183250 37548535 PMC10811998

[14] Przyborowska P Adamiak Z Holak P Zhalniarovich Y Maksymowicz WS. Diagnosis of cerebral ventriculomegaly in felines using 0.25 Tesla and 3 Tesla magnetic resonance imaging. Vet Med. (2018) 63:1–8. doi: 10.17221/59/2017-VETMED

[15] Przyborowska P Adamiak Z Zhalniarovich Y. Quantification of cerebral lateral ventricular volume in cats by low-and high-field MRI. J Feline Med Surg. (2017) 19:1080–6. doi: 10.1177/1098612X16676434 27827800 PMC11110985

[16] Pedersen NC Perron M Bannasch M Montgomery E Murakami E Liepnieks M . Efficacy and safety of the nucleoside analog GS-441524 for treatment of cats with naturally occurring feline infectious peritonitis. J Feline Med Surg. (2019) 21:271–81. doi: 10.1177/1098612X19825701 30755068 PMC6435921

[17] Doenges SJ Weber K Dorsch R Fux R Fischer A Matiasek LA . Detection of feline coronavirus in cerebrospinal fluid for diagnosis of feline infectious peritonitis in cats with and without neurological signs. J Feline Med Surg. (2016) 18:104–9. doi: 10.1177/1098612X15574757 25736448 PMC11149007

[18] Felten S Hartmann K. Diagnosis of feline infectious peritonitis: a review of the current literature. Viruses. (2019) 11:1068. doi: 10.3390/v11111068 31731711 PMC6893704

[19] Schmidt MJ Kampschulte M Enderlein S Gorgas D Lang J Ludewig E . The relationship between brachycephalic head features in modern Persian cats and dysmorphologies of the skull and internal hydrocephalus. J Vet Int Med. (2017) 31:1487–501. doi: 10.1111/jvim.14805 28833532 PMC5598898

[20] Przyborowska P Adamiak Z Jaskolska M Zhalniarovich Y. Hydrocephalus in dogs: a review. Vet Med. (2013) 58:1–8. doi: 10.17221/6698-VETMED

[21] Polis B Polis L Nowosławska E. Surgical treatment of post-inflammatory hydrocephalus. Analysis of 101 cases. Childs Nerv Syst. (2019) 35:237–43. doi: 10.1007/s00381-018-4022-4 30564912 PMC6351511

[22] Dai X Qiao Y Wang B. Hydrocephalus secondary to COVID-19 infection. QJM. (2023) 116:559–62. doi: 10.1093/qjmed/hcad043 36944269

[23] Vasconcelos TDMF Nóbrega PR Ferreira GDM de Souza MLP Vanderlei AS de Castro JDV . Normal pressure hydrocephalus associated with COVID-19 infection: a case report. BMC Infec Dis. (2022) 22:216. doi: 10.1186/s12879-022-07184-x 35241017 PMC8892823

[24] Laubner S Ondreka N Failing K Kramer M Schmidt MJ. Magnetic resonance imaging signs of high intraventricular pressure-comparison of findings in dogs with clinically relevant internal hydrocephalus and asymptomatic dogs with ventriculomegaly. BMC Vet Res. (2015) 11:181. doi: 10.1186/s12917-015-0479-5 26231840 PMC4522113

[25] Chari A Karponis D Craven CL Khan AA Thorne L. Disproportionately large communicating fourth ventricle: pearls for diagnosis and management. Cureus. (2018) 10:1–10. doi: 10.7759/cureus.3547 30648079 PMC6324856

